# Prototype theory and the importance of literary form for moral imagination

**DOI:** 10.3389/fnhum.2024.1329628

**Published:** 2024-04-11

**Authors:** Yi Zheng

**Affiliations:** Department of English Language and Literature, Hong Kong Baptist University, Hong Kong, Hong Kong SAR, China

**Keywords:** prototype theory, moral imagination, literary form, Martha Nussbaum, Henry James, *The Golden Bowl*

## Abstract

Prototype theory, which argues that categories have graded (and thus fuzzy) membership based on prototypes, has been used as cognitive evidence to support moral particularism because if categories (in moral rules) only have fuzzy conceptual boundaries, moral rules are not enough for moral judgment, as specific situations also need to be considered to determine how these fuzzy categories should be understood, which is what moral particularism believes. The importance of literature for ethics, especially for moral imagination, has also been extensively discussed because literature can provide vivid examples for us to imagine different moral dilemmas, the consequences of different moral choices, and the feelings of different people facing different situations. Martha Nussbaum specifically argues that the literary form is the only adequate form to imagine certain complex moral situations. By analyzing concrete literary examples as well as the related ethical discussions and empirical findings, this article argues that, building on Nussbaum’s argument, prototype theory can serve as a cognitive basis for the importance of literary form for moral imagination, because the literary form’s tolerance of ambiguity suits how we ambiguously categorize the world.

## 1 Moral particularism and its cognitive evidence

We are all familiar with the moral dilemmas that cannot be easily solved by universal moral rules, especially when two or more such rules conflict with each other in a particular situation: “Do not kill” and “minimize harm” conflict in the trolley problem, where switching the trolley to the track to which only one person is tied violates the former rule and doing nothing and letting five people tied to the current track die violates the latter. “Do not steal” and “save life” conflict in the Heinz dilemma, where stealing the drug violates the former and doing nothing and letting the wife die violates the latter. Moral particularists thus argue that no moral rules can be indiscriminately applied to every situation and that what is moral should be determined case by case (Leibowitz, 2013; Dancy, 2017; Ridge and McKeever, 2023). Emphasizing the significance of context and the complexity of individual moral situations, Aristotle is a forefather of moral particularism. He famously contends that to lead a good life, we should aim for an ideal intermediate state between the excess and the deficiency. What counts as intermediate (e.g., “When is it good to be angry and how much angry?”), however, cannot simply be determined by universal moral rules and reasoning alone but also by particular situations and perception (Taylor, 1990; Aristotle, 2009, pp. 1109b11–23, 1126b2–4).

In addition to traditional theoretical philosophical debates, moral particularists also incorporate empirical research from cognitive science as part of their arguments. Mark Johnson, for example, refers to Eleanor Rosch’s prototype theory of concepts (Rosch, 1973, 1975; Johnson, 1993). Rosch discovers in the 1970s that, for the question of how we categorize the world, our intuitive “definitional theory of meaning” does not hold. The definitional theory believes that our mental representations of word meanings are like the definitions of dictionary entries: When we decide whether an animal belongs to the concept “bird,” we recall a list of properties essential for membership in the bird category, such as “winged,” “covered with feathers,” “very likely being able to fly,” and “being able to lay eggs.” If the target animal fulfills these conditions, it is a bird regardless of its other traits such as how it looks or how common it is. However, Rosch finds that this is not the case and proposes a prototype theory instead: Rather than comparing the target animal with a list of properties necessary and sufficient for bird membership, what we do cognitively is to compare it with a “typical” bird, a prototype, to see how similar they are. The more similar they are, the more *likely* we would see the target animal as a bird. In other words, according to the definitional theory of meaning that has no grey area, a target animal is either a bird or not a bird, the only criteria being the properties of a clear definition of bird. According to Rosch’s prototype theory of meaning, however, the category word “bird” does not define clear boundaries but a centered, most fitting prototype. As a result, it has a *graded* membership depending on the similarity of a target animal to this prototype. The prototype theory explains why, in ordinary people’s understanding, some birds are “birdier” than other birds, although all the birds being compared meet the dictionary definition of bird. That is to say, when considering our cognitive process of daily language and not ornithology, whether an animal is a bird is not a strict either/or question, but a question of likelihood.

Over the years, from different perspectives and using different methods, a series of experiments have consistently demonstrated that most of our mental concepts indeed have a graded membership. The less similar a target concept is to the prototype—the further it is from the “center”—it is less likely to be thought of in a production task (“Name as many birds as you can”), requires more time to be processed semantically, and gets a lower score in a rating task (“Which bird is ‘birdier?’”). Robin and sparrow, for example, are far more “privileged,” i.e. “birdier,” than ostrich and penguin for the bird category. It does not matter much that they all have feathers and wings and lay eggs, thus all technically fit the definition of bird. This goes for concepts other than bird as well (Malt and Smith, 1984; Reisberg, 2019, pp. 329–332).

The prototype theory reminds us of Wittgenstein’s family resemblance theory, arguing that a group of instances may form a category even though no single feature common to all the instances exists: It is sufficient that each instance shares at least one feature with at least one more other instance. Indeed, many psychologists agree that most of our concepts have such a family resemblance structure (Medin et al., 1987; Gallagher, 2004, p. 166; Gleitman et al., 2011, pp. 413–414). To take Wittgenstein’s example, although board games, card games, ball games, and athletic games are all called games, we can only find an overlapping set of features shared by some of them (entertaining, competitive, etc.) but not a single feature that is common to all of them (many professional athletes do not find their games entertaining but regard them as jobs; single-player card games are not competitive). A clear, all-inclusive definition of “game” does not exist. Instead, we have “a complicated network of similarities overlapping and criss-crossing” (Wittgenstein, 2009, pp. 65–57; Family Resemblance, 2023).

Admittedly, not all concepts are like “game” and many concepts do have unambiguous definitions, such as “even number,” but in more cases, the definitions available are only generally true and exceptions are not difficult to find: a bird must have feathers, but a baby bird without feathers is still a bird; a chair is for sitting, but a chair covered with nails as an item on display at an art exhibition is still a chair. Psychologists since Rosch have demonstrated that our mind has a remarkable capacity to make such nuanced judgments. When this capacity of conceptual organization operates, it does not always follow rules and definitions, even if they are as well-defined as, for example, “even number.” In a peculiar, widely cited experiment designed by Armstrong et al. (1983), participants are asked to rate “how even” a list of numbers are, although they can all be exactly divided by two and thus are all by definition equally “even.” Interestingly, some even numbers, such as 2, 4, 8, or 1,000 are regarded as much “evener” than others, such as 34 or 106. This suggests that concepts like even number, a mathematically strictly defined concept that seemingly leaves absolutely no room for ambiguity, can also be represented cognitively as having graded membership, organized around a prototype (Reisberg, 2019, p. 338). While we may argue that when forced to make mathematically absurd judgments, the participants may intentionally or unintentionally add other features like “more common” or “easier to calculate” to the feature “evener” (therefore the result), this can happen in the cases of other concepts as well and it still shows how “irrational” and unruly our judgment can be.

To briefly summarize, we have now discussed that (a) most of our everyday concepts do not have clear-cut definitions but a family resemblance structure; (b) we might have traditionally and dogmatically been too obsessive about definitions, because we actually organize conceptual knowledge around prototypes rather than dictionary-definition-like lists of features; (c) as a result of being organized around prototypes, these concepts only have graded membership and fuzzy boundaries, whose determination requires judgment call.

Here is where moral particularists include the prototype theory as supporting evidence for their argument. We recall that moral particularists believe that each moral situation is unique and requires its own individual moral judgment. They point out that the concepts involved in a moral question or a moral principle are not exceptions to Rosch’s discovery (Johnson, 1993, pp. 78–107), because these concepts also usually only have ambiguous definitions, admit flexible graded membership, and thus challenge the views that restrict morality to a system of universal, inflexible rules (Schwartz and Sharpe, 2006, p. 387). Schwartz notices that such conceptual structure is precisely what ethics need, because moral principles as rigid as “Be loyal” or “Tell the truth” are not always helpful, as they fail to recognize the complex situations where being loyal or telling the truth might not be the best idea (Literature, as we will see in the following sections with the help of Henry James’s *The Golden Bowl*, is therefore a better form to vividly reproduce and insightfully explore such concrete situations). While the fuzzy nature of categorization around prototypes certainly makes it difficult to navigate in the moral world, our experience tells us that this is how real life is and why practical wisdom is indispensable.

To further emphasize the importance of practical wisdom in categorization, Schwartz elaborates on how arbitrary a category can be formed. We recall that judgment of similarity plays a fundamental role in conceptual organization because we put a target item into a category not by checking it against a definition but by determining how much it resembles the typical item of that category (the prototype). Such judgment of similarity seems straightforward, but consider this example: Are a plum and a lawn mower similar? While our experience makes us focus on their differences, Schwartz points out that “they are both found on earth, they both weigh less than a ton, they can both be dropped, they both cost less than $1,000, they are both bigger than a grape” (Schwartz and Sharpe, 2010, p. 58). The last similarity of this bizarre list is perhaps the most outrageous and surprising, as “grape” can be substituted by anything smaller than a plum and a lawn mower, which is a convincing example of how tricky and flexible judging similarity and forming categories (e.g., “things bigger than a grape”) can be. Both in moral life, as we have mentioned before, and in literature, as we shall find out later, an ability that is as flexible as this when taking the concrete situation at hand into account, which goes beyond fixed rules (often unconsciously), plays a decisive role. It is worth noticing that moral particularists do not suggest that we do not need moral principles at all. On the contrary, clear and definite rules are essential; what they do argue is that rules alone are not enough, because discerning the ambiguous concepts in the rules and deciding how the rules should be applied requires practical wisdom.

## 2 The moral significance of literature (in general)

We are also familiar with how closely related literature and moral philosophy are, especially in terms of moral imagination. Aristotle’s influential dictum, “the poet’s task is to speak not of events which have occurred, but of the kind of events which *could* occur” (Aristotle, 1987, pp. 40, 1451a36–38), emphasizes this relation. Moral imagination is so important that it can be characterized as the basic way we learn about morality, because even moral philosophers who are not moral particularists admit that learning about morality involves the ability to conceive ideas, metaphors, and situations that go beyond abstract moral rules and concrete direct observation. In literary studies, proponents of ethical criticism such as F. R Leavis, Wayne Booth, Noël Carroll, Cora Diamond, and Martha Nussbaum are particularly fascinated by questions like how literature that can be regarded as morally relevant is imagined, how literature in turn stimulates moral imagination, and how such imagination helps us making responsible real-life moral decisions.

Diamond (1995, pp. 299–300), for example, cites Dickens’s imaginative yet realistic description of how the world looks like to Pip, a helpless orphan and the protagonist in *Great Expectations*, from the child’s own perspective: “I remember Mr. Hubble as a tough high-shouldered old man, of a sawdusty fragrance, with his legs extraordinarily wide apart: so that in my short days I always saw some miles of open country between them when I met him coming up the lane” (Dickens, 2008, p. 23). She argues that this does a much better job of moral education than simply telling the reader that children see the world differently in a way that deserves adults’ attention and respect. Dickens indeed strives to evoke empathy towards children through such imagination, with many aspects of his novels reflecting this intention and its moral significance. By encouraging the reader to view the world through a child’s eyes and recognizing the unique way children perceive and interpret the world (“I always saw some miles of open country between them”), Dickens invites us to put ourselves into situations such as being ignored, experiencing unjust treatment, or intrusions into one’s privacy. More importantly, he does this not by simply exposing the reader to previously unknown facts with terse statements or moral lectures, but by inducing and embodying a concrete emotional response enabled by moral imagination. Such imagination makes his literary focus on children’s lives and thoughts characterized by warmth, humor, concentrated energy, and a distinctive style of affectionate curiosity towards human affairs (Diamond, 1995, pp. 299–300).

Nussbaum (1990) also generally argues that, first, moral judgment directly entails moral imagination, because when we encounter a moral dilemma, we inevitably imagine what a judge or a legislator would do in the same situation. She notices that, in the legal community, good legal judgment is increasingly being seen as Aristotle sees it, namely as how the perception of particular, concrete situations supplements the generalities of written, universal laws. Second, because such particular, concrete situations can be richly represented by certain novels with thick enough descriptions and realistic enough settings, such as Henry James’s, these novels have profound moral significance (Nussbaum, 1990, pp. 100, 166).

However, this article does not just state that literature is *sufficient* for better imagining morality by providing fictional yet possible examples, but also that the literary form (based on its tolerance of ambiguity) is *necessary* for moral imagination in many cases, and that this argument can be supported by empirical research. This argument builds on Nussbaum’s influential theory on the moral significance of literature, and this empirical support refers to the prototype theory and its experimental evidence mentioned above.

## 3 Nussbaum’s theory on the moral significance of literature

Implicitly referring to “winged words,” a wonderfully imaginative Homeric formula, Nussbaum argues that “the terms of the novelist’s art are alert winged creatures, perceiving where the blunt terms of ordinary speech, or of abstract theoretical discourse, are blind, acute where they are obtuse, winged where they are dull and heavy” (Nussbaum, 1990, p. 5). She believes that because the distinction between morally good and bad decisions lies in getting the particular situation right, only a form dedicated to a fine rendering of real moral life’s particularity and complexity can adequately serve moral philosophy. The literary form, especially that of a narrative artist, is such a form. Henry James’s *The Golden Bowl*, for example, shows why only language as dense, concrete, and subtle as the one in this novel can properly discuss certain moral questions.

Published in 1904, *The Golden Bowl* is James’s last major work, featuring its irreducible style and sharp attention to detail of the complicated interrelationships between its four main characters. The novel begins as Maggie Verver, daughter of a wealthy American widower Adam Verver settling in London, marries an impoverished Italian prince, Amerigo, without knowing that he and her best friend, Charlotte Stant, had been lovers (They could not afford marriage because of their mutual poverty). Before Maggie’s wedding, Amerigo and Charlotte go to an antique shop for a wedding gift. They find an interesting, gilded crystal bowl but choose not to buy it because Amerigo believes that the bowl has a crack.

Maggie and Adam always had a special bond. However, after Maggie has married, she finds that she and her father are not as close as before. Fearing that Adam could feel isolated and lonely, Maggie convinces him to marry Charlotte. Adam agrees. Afterward, the father and the daughter continue to spend most of their time together, even at the cost of leaving their respective spouses out in the cold. As Charlotte and Amerigo are left attending social events together, they restart their old relationship.

Although Maggie suspects they may have an affair, she has no solid proof. It is only when she happens to buy the same golden bowl that once attracted Charlotte and Amerigo that Maggie learns from the shopkeeper that her husband and her best friend seemed in love when they spoke to each other in the shop. She confronts Amerigo but he seems unmoved. Then, by tactfully convincing her father to return to America with Charlotte, Maggie successfully drives Amerigo and Charlotte apart without telling Adam or Charlotte what she knows. Impressed by Maggie’s tact, Amerigo, who had only thought of Maggie as a naive girl, has new feelings for her. At the end of the novel, Amerigo professes his love to Maggie by saying that he can see nothing but her, and the two embrace.

James, in agreement with moral particularists, regards moral judgment not simply as judgment based on general rules but as perception of particulars, emphasizing the moral significance of taking in all the details and nuances of the relevant situation. Importantly, such perception involves putting oneself in someone else’s position with imagination. Literature can both train us to do so and offer us a form to provide a satisfactory description of the situation itself. Therefore, James’s novels are morally interesting not because they happen to be so, but because the author is acutely conscious of what he is doing. Mitchell (2003) notes that although *The Golden Bowl* extensively discusses many moral questions, the text itself is irreducible to explicit moral rules. By saying this, Mitchell does not want to suggest that James is being willfully obscure, but “to register the supreme imaginative restlessness of his work.” As a result, “to convert unsettling language into a series of settled ideas […]—of rules, exemplars, models, allegories, or the like—is to miss James’s achievement in order to domesticate him to our own uses” (Mitchell, 2003, p. 87).

It is important to stress that Nussbaum’s argument for the moral significance of literature not only says that literature provides illuminating examples or literature creates possible worlds for moral philosophy, but also that, for moral discussion, what is said and *how* it is said are inseparable because certain morally significant aspects are so nuanced and context-specific that they cannot be adequately captured in a plain summary or paraphrase. Only literature, according to Nussbaum, especially novels that constantly strive to describe particular situations as comprehensive and realistic as possible, can deliver enough details necessary for responsible moral judgment, which needs these details to determine what rules are relevant, how exactly they should be applied, or even to revise the existing rules according to the current case.

As many critics point out, the plot of *The Golden Bowl*, compared to the other pieces commonly included in the discussion on literature and ethics, is rather straightforward and undramatic. In David Brudney’s words: “Almost nothing happens. In the course of more than five hundred pages there are two marriages, one affair, and a single act of violence, the smashing of the golden bowl. The rest is reflection, nuance, detail” (Brudney, 1990, p. 397). However, it is exactly the reflection, nuance, and detail that make it relevant for our discussion. Zheng (2022, 99–163) discusses how this novel explores why rules like “Do not lie,” “Be considerate towards others,” “Be filial/grateful to parents,” and “Take good care of children” as well as general terms like “love,” “friendship,” “father,” and “daughter” do not suffice for good moral judgment. Literature matters because its dedication to details matters (and, more exclusively, as we shall see in the next section, because its tolerance of ambiguity matters).

Take “Be filial/grateful to parents” and “Take good care of children” for example, moral rules like these that include general terms such as “parents” or “children” often prove inadequate to be useful because they have different moral significance for different people in different situations. On the one hand, a simple “closed parent–child relationship” cannot fully describe the morally salient features of the situation between Adam and Maggie. On the other hand, as Nussbaum (1990, pp. 90–91) points out, neither does Adam, a “so remarkably distinct figure” (James, 1909, p. II.330), mean to Maggie only as an abstract “parent” nor can Maggie, who has been “more than a daughter” (James, 1909, p. I.134), be dogmatically fitted into any moral rules that dictate what should or should not be done to a child by a parent. For example, here is how Maggie tries to convince her father to get married:

“Should you really,” he now asked, “like me to marry?” He spoke as if, coming from his daughter herself, it *might* be an idea; which for that matter he would be ready to carry right straight out should she definitely say so.

Definite, however, just yet, she was not prepared to be, though it seemed to come to her with force, as she thought, that there was a truth in the connexion to utter. “What I feel is that there’s somehow something that used to be right and that I’ve made wrong. It used to be right that you hadn’t married and that you didn’t seem to want to. It used also”—she continued to make out—“to seem easy for the question not to come up. That’s what I’ve made different. It does come up. It *will* come up.”

“You don’t think I can keep it down?” Mr. Verver’s tone was cheerfully pensive.

“Well, I’ve given you by *my* move all the trouble of having to.”

He liked the tenderness of her idea, and it made him, as she sat near him, pass his arm about her. “I guess I don’t feel as if you had ‘moved’ very far. You’ve only moved next door.”

“Well,” she continued, “I don’t feel as if it were fair for me just to have given you a push and left you so. If I’ve made the difference for you I must think of the difference.”

“Then what, darling,” he indulgently asked, “do *you* think?”

“That’s just what I don’t yet know. But I must find out. We must think together—as we’ve always thought. What I mean,” she went on after a moment, “is that it strikes me I ought to at least offer you some alternative. I ought to have worked one out for you.”

“An alternative to what?”

“Well, to your simply missing what you’ve lost—without anything being done about it.”

“But what have I lost?” (James, 1909, pp. I.171–172).

Although this is only the beginning of a lengthy, awkward conversation, there are already many subtle nuances worth exploring. It begins with Adam kindly helping Maggie to say the things she had difficulty saying, namely suggesting that he should get married. Although surprised by her father’s straightforwardness, Maggie still manages to take up the thread. This has set the tone for who is the calm and active party and who is the nervous and passive party in this unpleasant encounter. Adam seems passive but is actually in control, while Maggie, who is supposed to be proactive, appears less confident when she struggles to hide her manipulative intention. By emphasizing that something wrong *will* come up, Maggie implies that Adam cannot handle the situation of being single in the long run, which is again straightforwardly brought out into the open by Adam. By being “cheerfully pensive,” Adam is considering something we do not know for sure. Is he a bit humiliated by the lack of confidence of his daughter for him (but pretends to be cheerful)? Or does he understand that Maggie only suggests this for his own benefit, at least in her mind? Maggie evades his question and points out that she feels guilty about having to move out after her own marriage. While this may not be a big deal for other fathers and daughters, it is for Maggie and Adam because they have always been particularly close for several reasons: (1) Maggie is the only child and Adam is a widower; (2) They both share a passionate interest in art; (3) Maggie has been protecting Adam from harm, such as shielding Adam from fortune-hunting women like Mrs. Rance. However, Adam’s reply seems indifferent (“I guess I do not feel as if you had ‘moved’ very far.”), although the narrator indicates otherwise by stating that he appreciates Maggie’s good intention. Maggie again evades Adam’s reply and insists on being considerate towards him and doing good to him on his behalf, as if Adam does not know what is good for himself. Although Adam has seen through her already at the beginning of this conversation and has taken the initiative to let her speak her mind directly, Maggie here again pretends as if she does not know exactly what she is suggesting (In fact, she is more than clear. She has even already chosen the candidate of her stepmother). By repeatedly asking what Maggie means, Adam not only shows a loving father’s excessive indulgence, but also almost practically makes fun of her. After all, how insensitive (or, indeed, stupid) does Maggie think Adam is so that he needs others to tell him what he has lost by being single?

So far, we have offered a summary of the cited passage with some additional background information. What would readers of this summary miss that can be significant for a responsible moral judgment of, say, Maggie’s action? They can of course get that here each party in this conversation treats the other as a child. Maggie, in particular, is trying to restrict his father’s freedom in the name of his best interest. What they would not get are, for example, *how exactly* reluctant Adam is when considering Maggie’s suggestion and *how exactly anxious but firm* Maggie is when persuading Adam. This is not to say that readers would all unanimously agree on the exact degrees of “how,” because ambiguity prevails (Is Adam as cheerful and tolerant as the narrator depicts?), but it does suggest that readers would get *more* (if not the whole picture) when reading the original full text. We are all aware that something must be missing when the original text is summarized or paraphrased, but it has usually been treated as an acceptable “necessary evil.” Even Nussbaum who argues that to judge Maggie we should quote the whole novel does not end up actually doing it (Nussbaum, 1990, p. 88). Is there anything wrong with this pragmatic attitude? Are there cases where the missing information is so important that, for a moral evaluation, the literary form is *necessary*? Let us resume the previous dialog:

She thought a minute, as if it were difficult to say, yet as if she more and more saw it. “Well, whatever it was that *before* kept us from thinking, and kept you, really, as you might say, in the market. It was as if you couldn’t be in the market when you were married to *me*. Or rather as if I kept people off, innocently, by being married to you. Now that I’m married to some one else you’re, as in consequence, married to nobody. Therefore you may be married to anybody, to everybody. People don’t see why you shouldn’t be married to *them*.”

“Isn’t it enough of a reason,” he mildly enquired, “that I don’t want to be?”

“It’s enough of a reason, yes. But to *be* enough of a reason it has to be too much of a trouble. I mean *for* you. It has to be too much of a fight. You ask me what you’ve lost,” Maggie continued to explain. “The not having to take the trouble and to make the fight—that’s what you’ve lost. The advantage, the happiness of being just as you were—because I was just as *I* was—that’s what you miss.”

“So that you think,” her father presently said, “that I had better get married just in order to be as I was before?”

The detached tone of it—detached as if innocently to amuse her by showing his desire to accommodate was so far successful as to draw from her gravity a short light laugh. “Well, what I don’t want you to feel is that if you were to I shouldn’t understand. I should understand. That’s all,” said the Princess gently.

Her companion turned it pleasantly over. “You don’t go so far as to wish me to take somebody I don’t like?”

“Ah father,” she sighed, “you know how far I go—how far I *could* go. But I only wish that if you ever should like anybody you may never doubt of my feeling how I’ve brought you to it. You’ll always know that I know it’s my fault.”

“You mean,” he went on in his contemplative way, “that it will be you who’ll take the consequences?”

Maggie just considered. “I’ll leave you all the good ones, but I’ll take the bad” (James, 1909, pp. I.172–173).

After Adam asks Maggie to enlighten him about what he has lost by being single, she continues to pretend that what she is doing is not planned beforehand. Maggie conveniently compares their closed relationship to marriage and attributes the reason why people are turned away from Adam to her “spousal” protectiveness. She adds, seemingly casually but deliberately, that whatever she does, either keeping people off or being “married” to her father, is done innocently, as if she cannot help it. Just when we assume that this incestuous metaphor cannot be serious, Maggie’s next argument indicates that she means it. According to her, the reason why people should not doubt Adam is available now is not that Maggie has realized her overprotection and will correct her mistake, but that people see Maggie is now *actually* married to someone and thus cannot be in the way. The fact that such an inappropriate metaphor can be thought of effortlessly and shamelessly without qualification by Maggie, and that it is not resisted by Adam illustrates not only the unusual intimacy between the father and the daughter, but also their appalling moral indifference and naivety. This should have a direct bearing on the moral consequences of their actions, both before and after Adam’s marriage, but readers who only read its paraphrase may fail to grasp its subtlety, to grasp just *how exactly* brazen Maggie is (and at the same time perhaps too childish to be harshly criticized) and *how exactly* almost unforgivably indulgent Adam is (and at the same time perhaps understandably). Indeed, at the end of this conversation, which is not cited in full here, Adam gives in.

A much simpler (and more common in practice) summary of what we have quoted so far from the beginning of this conversation would be: “Although Adam is satisfied with being single, he agrees to get married just to keep Maggie from worrying about him after several rounds of back-and-forth.” Only based on this, we may quickly judge Adam as irresponsible, especially to his future wife because he does not marry for romantic love but for her daughter. We may also think it is better for him to simply say no and explain that he is genuinely fine to Maggie. However, if we read the whole conversation, we will find that it is much more complicated than this. First, Maggie has firmly made up her mind and seems not to be persuaded. Second, her intention is arguably good, and it may break her heart to turn her down, considering their particularly intimate father-daughter relationship. Third, Adam’s decision to get married for her daughter is admittedly morally risky, but what if Adam and Charlotte actually can make a good couple even without Maggie’s meddling, considering that they have been getting along well anyway? Considering these may not eventually make a difference to our moral evaluation, but they are surely important. Maggie’s stubborn persistence, Adam’s futile resistance, and the heated confrontation between the two cannot be simply summarized by “several rounds of back-and-forth” either. Concrete words from Adam like “But *what* have I lost?,” “Isn’t it enough of a reason […] that I do not want to be?,” “You do not go so far as to wish me to take somebody I do not like?,” and “What do you want […] to do to me?” all matter for readers to grasp the nuances of what is at hand here. So do the detailed descriptions of Adam’s and Maggie’s mental activities, as our close reading above has shown. They together present us with a concrete, unique situation that defies simple paraphrasing and challenges an indiscriminating application of universal moral rules.

## 4 Prototype theory, the literary form’s tolerance of ambiguity, and its necessity for moral imagination

Despite the huge influence of Nussbaum’s theory on the moral significance of literature, both Nussbaum’s own argumentation and its academic reception leave something to be desired, such as answering what exactly makes the literary form necessary for moral philosophy (see Zheng, 2022, pp. 163–174). A cognitive perspective can help here by suggesting that it is ambiguity that makes the literary form important because this resembles how we cognitively categorize the world. We have discussed how most of our concepts have no clear-cut definitions and are organized around prototypes. Even for strictly defined concepts such as “even number,” people still, for whatever reasons, distinguish between “evener” numbers and “less even” numbers, convincingly showing how seemingly irrational our judgment can be.

However, such flexible nature of our conceptual organization with no clear boundaries serves responsible moral judgment well, because, as demonstrated by the previous sections, too rigidly defined concepts and rules fail to recognize the uncertainty and the unpredictability of the concrete situations as well as our conflicting obligations that are ubiquitous in real moral life. Consequently, if there is a form that allows such ambiguous conceptual organization, it is a naturally fitted form to describe real-life situations and how we cognitively categorize them. The literary form, by its commonly accepted definition, is such a form.

As Brudney (1990, p. 417) notices, seeing a literary text as morally philosophical faces the problem of how to deal with ambiguity, which is undesirable in philosophy but entirely acceptable, if not desirable, in literature. Because philosophy, to quote Wittgenstein, “aims at the logical clarification of thoughts” (Wittgenstein, 2001, p. 4.112), different interpretations of the same clarification are not welcome. Literature, however, lives on different interpretations. Rosch’s finding that our conceptual organization is just as ambiguous undoubtedly makes the literary form that tolerates ambiguity more worthy of study. The previous section briefly analyzes how untypical the relationship between Adam and Maggie is and how untypical the relationship between Charlotte and Maggie is (for detailed analysis, see Zheng, 2022). It also analyzes, from the perspective of literary studies, how such untypicality makes universal rules that contain general terms such as “parent,” “daughter,” or “friend” less useful, and how their application thus requires practical wisdom. This also makes particular sense when considered from the cognitive perspective. Because our conceptual knowledge is not based on dictionary-like definitions of concepts but prototypes of concepts, it is probabilistic instead of definite in the sense that the more a target item resembles the prototype of a concept, the more likely we are to judge it as belonging to that concept. In the case of *The Golden Bowl*, this explains why although what is between Adam and Maggie technically fits the definition of a “father-daughter relationship,” due to their unusual intimacy and all the other complications discussed earlier, we would hesitate to classify it into this concept because they barely resemble the prototype of father and daughter. Therefore, rigid rules with such only technically fit concepts are of little use, while moral imagination is always useful because of the wiggle room left by our ambiguous conceptual organization.

To find out more subtleties in *The Golde Bowl* only perceivable in the original literary form but not in the standard prose of a summary, consider how Adam proposes to Charlotte:

Every evening after dinner Charlotte Stant played to him; seated at the piano and requiring no music she went through his “favourite things”—and he had many favorites—with a facility that never failed, or that failed but just enough to pick itself up at a touch from his fitful voice. She could play anything, she could play everything—always shockingly, she of course insisted, but always, by his own vague measure, very much as if she might, slim sinuous and strong, and with practiced passion, have been playing lawn-tennis or endlessly and rhythmically waltzing. His love of music, unlike his other loves, owned to vaguenesses, but while, on his comparatively shaded sofa, and smoking, smoking, always smoking, in the great Fawns drawing-room as everywhere, the cigars of his youth, rank with associations—while, I say, he so listened to Charlotte’s piano, where the score was ever absent but, between the lighted candles, the picture distinct, the vagueness spread itself about him like some boundless carpet, a surface delightfully soft to the pressure of his interest. It was a manner of passing the time that rather replaced conversation, but the air at the end none the less, before they separated, had a way of seeming full of the echoes of talk. They separated, in the hushed house, not quite easily, yet not quite awkwardly either, with tapers that twinkled in the large dark spaces, and for the most part so late that the last solemn servant had been dismissed for the night (James, 1909, pp. I.202–203).

This episode happens not long after the one we have discussed, where Maggie has successfully convinced Adam to propose to Charlotte. Maggie and Amerigo then depart for Rome to see the Prince’s ancestral home, leaving Adam and Charlotte alone in London. During this time Adam certainly knows Charlotte better, but does he begin to love Charlotte? We can find some clues from this seemingly uneventful but psychologically penetrating passage that critics rarely talk about. Although these clues cannot give us a conclusive answer, they can help us grasp some morally significant nuances so that we can begin to responsibly evaluate the situation. The narrator first adopts a quasi-neutral perspective to describe Charlotte’s musical talent. Both what she plays and how she plays please Adam, but he neither loves music as much as he loves collecting art, nor does he know music as much. Adam thus somewhat only uses her music as an ideal background for deliberation, a tool for creating a comfortable atmosphere. Although the passage reads peacefully, we can catch a glimpse of Adam’s stressfulness in the metaphor of the boundless carpet, where the vague atmosphere created by Charlotte’s music is compared to “a surface delightfully soft to the pressure of his interest”: He is under the pressure of Maggie’s suggestion to marry Charlotte. Importantly, the narrator points out that although Adam and Charlotte are not talking directly, they are still communicating tacitly. It is unclear if Charlotte knows what Adam is thinking about. If so, such tacit mutual understanding would be significant progress in their relationship. It is also unclear if Adam truly falls in love with Charlotte. If so, such mutual understanding would become mutual admiration and form a morally irreproachable foundation for marriage. Then neither is Adam marrying Charlotte only to reassure Maggie nor is he making use of Charlotte without considering how she feels, which obviously makes all the difference for our moral evaluation of this situation. Because a simple summary of this passage cannot fully express this ambiguity, a moral evaluation based on it would be irresponsible.

What is then Adam’s final decision?

The sharp point to which all his light converged was that the whole call of his future to him as a father would be in his so managing that Maggie would less and less appear to herself to have forsaken him. And it not only wouldn’t be decently humane, decently possible, not to make this relief easy to her—the idea shone upon him, more than that, as exciting, inspiring, uplifting. […] The way in which it might be met was by his putting his child at peace, and the way to put her at peace was to provide for his future—that is for hers—by marriage, by a marriage as good, speaking proportionately, as hers had been. As he fairly inhaled this measure of refreshment he tasted the meaning of recent agitations. He had seen that Charlotte could contribute—what he hadn’t seen was what she could contribute to. When it had all supremely cleared up and he had simply settled this service to his daughter well before him as the proper direction of his young friend’s leisure, the cool darkness had again closed round him, but his moral lucidity was constituted. […] He might have been equally in want and yet not have had his remedy. Oh if Charlotte didn’t accept him the remedy of course would fail; but, as everything had fallen together, it was at least there to be tried. And success would be great—that was his last throb—if the measure of relief effected for Maggie should at all prove to have been given by his own actual sense of felicity. He really didn’t know when in his life he had thought of anything happier. To think of it merely for himself would have been, even as he had just lately felt, even doing all justice to that condition—yes, impossible. But there was a grand difference in thinking of it for his child (James, 1909, pp. I.207–209).

This passage presents us with a still-water-runs-deep person. We finally fully see what intricate considerations lie behind the man who smokes silently and stressfully night after night to Charlotte’s music. Just when we, based on the piano passage, begin to suspect that Adam has real feelings for Charlotte, this passage makes it again clear that the only thing he cares about is still how not to make Maggie feel guilty and not to make her feel like she has abandoned him. It seems that the ambiguity of whether Adam selfishly objectifies and exploits Charlotte can be finally resolved: Not only does he see that “Charlotte could contribute,” but he also believes that he is entitled to determine “the proper direction” of her leisure. However, newly emerging ambiguities suggest that the case is not so simple. First, if Adam conscientiously believes that it is moral to solve his problem by simply making use of Charlotte, why does he feel that he is again surrounded by “cool darkness?” This indicates that Adam may still worry that by doing so he will wrong Charlotte, despite his constituted “moral lucidity.” Second, the narrator leaves us enough reasons to suspect that, to make himself look like a great father, Adam may be lying to himself that the only reason he marries Charlotte is to make Maggie worry less. He is so self-absorbed in his image as a self-sacrificing father and his concern for Maggie that the idea to relieve Maggie shines on him not only as “decently humane, decently possible” but also as “exciting, inspiring, uplifting.” The narrator’s excessive enumeration of adjectives sounds mechanical and suggests sarcasm. Adam’s closing thoughts, which have been taken by many critics at face value, actually also make him too noble to be trusted. How can the happiest thing in his long life be to marry someone he does not truly love to reassure his daughter? We can thus reasonably doubt that putting Maggie’s mind at ease is at least not the *only* reason Adam decides to marry Charlotte; he marries her also because of love. What we do not know is the proportion of the influence of each of these two reasons on his final decision. Considering that Adam goes so far as to claim that thinking merely for himself is “impossible” (by contrast, he can do anything for his child), it is likely that he exaggerates the role of being considerate towards Maggie and downplays the role of his love for Charlotte. For a moral judgment, the greater the role of the former objectifying reason is, the more morally reprehensible Adam’s decision is. It is therefore morally responsible to fully grasp both the direct portrayal of Adam’s mind here and the more lyrical portrayal of his silent communication with Charlotte discussed above and to compare them. We again see that a literary form that can reflect highly ambiguous complexity significantly contributes to understanding this concrete situation.

Brudney (1990, pp. 415, 434) insightfully points out that Maggie’s extraordinary courage is best displayed not by her willingness to suffer “for love” but by her willingness to endure high *ambiguity*, which preserves the last hope of her marriage. Maggie does not definitely know the answers to any of the following questions: Does Amerigo love her? Does he love Charlotte? Does he sincerely come around or is he only being perfunctory? Neither do we, because James does not want to use his author’s privilege to give us any direct access to Amerigo’s inner world. This obviously creates ambiguity but also, perhaps more importantly, authenticity because this is how real moral life looks like (We do not have direct access to the inner world of any real person either), and, thanks to the findings of cognitive science, we now know that the way how we categorize the world is as ambiguous.

Johnson (1993, p. 91–93) offers another powerful example of the relevance of the prototype theory for moral imagination. As Coleman and Kay (1981) and Sweetser (1987) show, the existence of white lies, social lies, mistakes, jokes, exaggerations, and oversimplifications makes defining lies notoriously difficult. There are three core conditions for a lie: the speaker believes it to be false; the speaker intends to deceive; and the content is indeed false. Instances that fulfill only one or two of these conditions are non-typical lies. In particular, the third condition (whether the content is indeed false) is actually the least important condition in the definition of a lie, since the contents of almost all non-typical lies that would make people hesitate to call them lies are false (“You look beautiful”; “The stress is killing me;” “I’m on a whiskey diet”). Yet, the condition of a lie that first comes to the mind of most people is this very least important one. This is again strong evidence that we do not judge whether a statement is a lie or not based on a clear, dictionary-entry-like definition, but rather on how much the statement resembles the typical lie in our minds, which is obviously a highly ambiguous process.

We have mentioned that, for readers who only read a summary of the original literary text, in addition to the obvious loss of details of the story, they lose the opportunity to grasp the ambiguities of the situation, which is regarded as undesirable by many forms other than the literary one. The prose of analytical philosophy, for example, aims to eliminate ambiguities. However, as discussed, both in fiction and in real life, the situation is often ambiguous because we often have to infer what really happens from what is only ostensibly there. As a result, not only can we disagree about what rules should be applied and how, but we can also disagree about what the facts are and how they should be interpreted. We have offered several examples where our moral judgment will be significantly different if a detail of the situation is left out or a word is formulated in other ways. Thus, although our discussion is based on a work of fiction, it is reasonable to believe that, even when discussing moral issues that actually occur, a form that allows ambiguity is important for fully grasping the situation so that responsible moral judgment can be made. As a result, a plot summary, with its nonliterary form, does not work for the purpose of ethical discussion.

*The Golden Bowl* can tell us both “A daughter should do what Maggie does in the exact case where the daughter is like Maggie and the father like Adam,” and “A daughter should, like Maggie, consider all aspects of the moral dilemma she encounters.” Both of these “rules” can be applied universally. However, such universalized statements are not real moral rules, but merely directions for moral imagination, because moral rules as specific as the former statement have little scope for application, while moral rules as general as the latter are just platitudes. While James often stresses the analogy between the work of the moral imagination and the work of the creative imagination, especially that of a novelist, Nussbaum sees beyond this analogy and wants to investigate how moral imagination finds in novels its most appropriate articulation (Nussbaum, 1990, p. 148). She critically notes that because of the high quality and intensity of Adam’s moral imagination, which is subtle, rich, and loving, how he sees his daughter’s sexuality and their father-daughter relationship can be captured linguistically only in the literary form: to morally assess this situation fully, its specificity must be fully considered as well. Condensed statements like “Adam thought of Maggie as an autonomous being,” or “He acknowledged his daughter’s mature sexuality,” or even “He thought of his daughter as a sea creature dipping in the sea,” which already seems like an acceptable compromise because of its inclusion of a key original metaphor by James, are not enough because we would miss the morally essential nuances mentioned above (Nussbaum, 1990, p. 152).

Both Aristotle and James believe that moral judgment is akin to perception, which is about intuitively grasping the ambiguous concrete situation at hand rather than rational moral reasoning (James, 1908, pp. I.vii–viii, xii–xiii; Aristotle, 2009, pp. 1142a23–31). Jonathan Haidt, in his groundbreaking study on the empirical foundation of moral judgment, uses almost the same wording: “When people grasp [moral truths] they do so not by a process of ratiocination and reflection but rather by a process more akin to perception” (Haidt, 2001, p. 814). Few scholars have addressed what such an empirical foundation means for the moral importance of the literary form. If responsible moral judgment dictates that none of the details of a concrete situation can be left out, then, when talking about moral education, we also need an appropriate form that allows us to fully and legitimately imagine all of these details. Considering that the concrete situations of everyday life are often full of ambiguities and, more importantly, that, according to prototype theory, the way we know the world is full of ambiguities at the boundaries of the concepts we create, it is evident that finding a form that allows for such ambiguities is essential.

James notes that it is essential “to imagine […] the honorable, the producible case” because “what better example than this of the high and the helpful public and, as it were, civic use of the imagination?—a faculty for the possible fine employments of which in the interest of morality my esteem grows every hour I live” (James, 1907, p. 223). Indeed, such moral imagination plays an important role in *The Golden Bowl*. A turning point in the novel is when Maggie begins to realize her mistake of leaving her husband out in the cold and being too close to her father, and after realizing this she grows up and begins to put herself in other people’s painful situations. For example, after Maggie confronts Amerigo about his cheating, Amerigo does not tell Charlotte about it and the latter thus becomes isolated. At this point, Maggie imagines her as “a prisoner looking through bars” (James, 1909, p. II.230). Towards the end of the novel, Maggie also imagines the Prince as a trapped animal in his study. It is such moral imagination that eventually leads Maggie to make the final wise decision to avoid a head-on confrontation so that everyone can save face. In a widely cited study directly named *The Ambiguity of Henry James*, Samuels (1971, p. 216) argues that such imagination exonerates Maggie from the charge of merciless justice because she can feel sympathy for her rival. Maggie’s imagination is contrasted by Charlotte’s assertion that “I cannot put myself into Maggie’s skin—I cannot, as I say. It’s not my fit—I should not be able, as I see it, to breathe in it” (James, 1909, p. I.311). This is further highlighted by the fact that in Maggie’s mind, Charlotte is not as bad: “I may be as good, but I’m not so great—and that’s what we are talking about. She has a great imagination. She has, in every way, a great attitude. She has above all a great conscience” (James, 1909, p. I.181; see also Zheng, 2022, pp. 122, 143, 150).

## 5 Discussion

On the one hand, moral particularists, who believe that moral judgment is not merely the mechanical application of moral principles to particular situations but includes nuanced fine-tuning based on the particular situations, have already brought the prototype theory, which argues that our concepts are mentally organized and processed around prototypes rather than clear definitions like dictionary entries, into moral philosophical debates. Contrary to the classical theory of category structure, which does not acknowledge the internal structure of individual categories but regards every member of the same category as the same, the prototype theory argues that some members are cognitively more central. When we try to determine whether an animal is a bird, for example, we usually do not compare it with a list of features defined by ornithology. Instead, we compare it with a prototype, a typical member of the category bird that is perhaps most common and therefore can be instantly thought of, such as a robin. In other words, the prototype theory points out that linguistic categories are fuzzy at the edges but clear in the center (Geeraerts, 2010, p. 183). Johnson (1993, pp. 8–9) thus concludes that if the basic concepts in moral principles are as ambiguous, moral principles alone are clearly not enough for sound moral judgment, because relying on them alone we cannot tell what principles apply to what situations. This ambiguity may dangerously give rise to moral relativism, but it does correspond to our everyday experience, and it also has the advantage of being able to keep the core of the moral principles intact while fitting new cases into it.

On the other hand, both literary scholars and philosophers have also been linking literature and moral philosophy. Some even argue that literature did not become an aesthetic category until the 19th century, and before that literature primarily belonged to the ethical sphere (Van Peer, 1995, p. 277). Moral concepts, they believe, because of their firm cultural embeddedness in narrative, can be very meaningfully discussed in literature. In particular, literature offers us a playground for moral imagination, which is crucial because many aspects of moral philosophy involve “what ifs.” Consequently, Nussbaum argues that moral particularism is best expressed not in the philosophical but in the literary form.

However, few studies have linked cognitive evidence, literature, and moral philosophy altogether. This article discusses the importance of the literary form for moral imagination by introducing prototype theory, a promising cognitive finding that has not been systematically linked to literary studies, into the debate. This is promising because the cognitive evidence that our conceptual organization is ambiguous helps us answer Nussbaum’s unanswered question: What makes the literary form unique in its contribution to ethics? We argue that it is ambiguity because other forms usually require the author to at least reduce the uncertainty of meaning and, if not, to adequately explain why, while literature, be it prose, poetry, or drama, is automatically ambiguous and the author should have a clear consciousness writing that way. This is also the case both in real moral life and in our cognition, making literature particularly relevant for ethics because the inherent (and perhaps desired) ambiguity of the literary form resembles the ambiguity of our conceptual organization, which plays a vital role in how we behave and thus obviously in ethics as well. Although emphasizing the intrinsic ambiguity of literature is an old argument, especially by New Criticism (Empson, 1966), linking it with cognitive science and moral philosophy adds new significance.

Emphasizing particulars does not deny the significance of universals for moral judgment. The relationship between particulars and universals in moral philosophy is not either/or. Nevertheless, as Nussbaum points out, although in some cases the concrete situation at hand can be outlined by general terms without sacrificing its idiosyncrasies, in other cases general terms cannot even outline the concrete situation in a morally significant way. By briefly analyzing Henry James’s classic novel, *The Golden Bowl*, we conclude that it is indeed the literary form that enables James to play with the ambiguity that richly represents the original (fictional but possible) situation, because many such situations are so nuanced that they truly require the ambiguity of literature to be faithfully depicted. We pay particular attention to the cases where the concepts of, for example, “father” and “daughter,” which can be easily included in moral rules, have different meanings for different people in different situations. It is thus unwise to blindly deny such complexity and dogmatically impose universality by saying that we should always behave so and so to our father or our daughter, regardless of the concrete situation. Critically, we dig into the ambiguous details of these cases and argue that they indeed often can only be comprehensively depicted by the literary form, which, unlike the other forms, inherently tolerates (if not champions) ambiguities (for further discussion, see Zheng, 2022, pp. 164–169).

Like Gadamer (2013), Nussbaum tends to entrench the already very deep division between the human and the natural sciences by arguing that her literature-matters-for-ethics argument proves that the unambiguous form favored by scientists does not always prevail, without realizing that ambiguity in cognition is not only recognized but a popular research topic in cognitive science. In this regard, this article also aims to demonstrate that this division does not have to be this deep and that cognitive poetics can be a two-way street where both literary scholars and scientists can learn from each other.

In a recent study (Hogan, 2022, p. 70), Patrick Colm Hogan, a pioneer of cognitive poetics, briefly touches on the interconnection among the flexibility of our narrative capacities, the trade-off between particularity and generality in (moral) cognition, and the ambiguity of categorization, but there is a lot more that can be done for humanities scholars. For future research, from the philosophical and literary perspective, we may further study the argument that the tolerance of ambiguity makes the literary form important for ethics in the context of several other established subfields, such as the study on ambiguity itself, on description and representation, on form vs. content, and on universals and particulars. In addition, associating Nussbaum’s argument with the well-developed stylistics, especially the studies on philosophical style, can be interesting (See, for example, Horton, 1996, p. 74). The question of literary interpretation also seems to be inseparable from metaphor theory (how to construct and understand secondary meanings beyond the primary ones), which has already witnessed fruitful conversations across the “two cultures” (See Holyoak, 2019, p. 658).

In another influential work on cognitive poetics, published in the current venue, Burke (2015) characterizes his article as “part critical review, part methodological proposal and part opinion paper,” which is also how this article sees itself. Similar to Burke’s aim, we aim to excite thinking in interdisciplinary research on literature, ethics, and cognitive science and to point to very concrete opportunities where humanities scholars and scientists may work together. Emphasizing moral imagination not only emphasizes the acknowledgment of the uniqueness of every individual and situation, but also that in order to responsibly make moral judgments, we should envision, as best as we can, all possible consequences for this particular individual and situation at hand. In this regard, acting morally requires more than just virtues and moral knowledge but the ability to mentally form new pictures and ideas, and cultivating this ability is certainly something that novelists, literary scholars, and cognitive scientists can all contribute to.

## Data availability statement

The original contributions presented in the study are included in the article/supplementary material, further inquiries can be directed to the corresponding author/s.

## Author contributions

YZ: Writing – original draft, Writing – review & editing.
